# Immune tuning scaffold for the local induction of a pro-regenerative environment

**DOI:** 10.1038/s41598-017-16895-0

**Published:** 2017-12-05

**Authors:** Bruna Corradetti, Francesca Taraballi, Claudia Corbo, Fernando Cabrera, Laura Pandolfi, Silvia Minardi, Xin Wang, Jeffrey Van Eps, Guillermo Bauza, Bradley Weiner, Ennio Tasciotti

**Affiliations:** 1 Department of Nanomedicine, Houston Methodist Research Institute, 6670 Bertner Ave., Houston, TX 77030 USA; 2 Department of Life and Environmental Sciences, Polytechnic University of Marche, via Brecce Bianche, 60131 Ancona, Italy; 3 Center for Biomimetic Medicine, Houston Methodist Research Institute, 6670 Bertner Ave., Houston, TX 77030 USA; 4 Houston Methodist Orthopedics and Sports Medicine, Houston, Texas, U.S.A.,, Houston, TX 77030 USA; 5 Center for Nanomedicine, Brigham and Women’s Hospital, Harvard Medical School, Boston, MA USA; 6 Center for NanoHealth, Swansea University Medical School, Swansea University Bay, Singleton Park, SA2 8PP Wales UK

## Abstract

In mammals, tissue regeneration is accomplished through a well-regulated, complex cascade of events. The disruption of the cellular and molecular processes involved in tissue healing might lead to scar formation. Most tissue engineering approaches have tried to improve the regenerative outcome following an injury, through the combination of biocompatible materials, stem cells and bioactive factors. However, implanted materials can cause further healing impairments due to the persistent inflammatory stimuli that trigger the onset of chronic inflammation. Here, it is described at the molecular, cellular and tissue level, the body response to a functionalized biomimetic collagen scaffold. The grafting of chondroitin sulfate on the surface of the scaffold is able to induce a pro-regenerative environment at the site of a subcutaneous implant. The early *in situ* recruitment, and sustained local retention of anti-inflammatory macrophages significantly reduced the pro-inflammatory environment and triggered a different healing cascade, ultimately leading to collagen fibril re-organization, blood vessel formation, and scaffold integration with the surrounding native tissue.

## Introduction

The ability to regenerate lost or damaged body parts is widespread among animal phyla and can vary intra and inter-species. Regenerative potential decreases with the increase of organism complexity and the capacity to fully regenerate the body is lost among adult vertebrates^1–4^. Urodels are able to efficiently and entirely regrow lost limbs, tail, jaws, and retina. The physiological reasons behind this peculiar ability are still controversial. Different hypotheses have been made, focusing either on the plasticity related to the developmental stage/aging of the organism^5^, to the role of the adaptive immune system, or to the local inflammatory response^6^. Certainly, this regenerative response involves the complex interaction between a variety of different cell populations and components of the extracellular matrices (ECM), usually organized in a structure called blastema^7^. It has been proposed that macrophages (Mϕ) are one of the key players in the process of regeneration. They are responsible for the initial reduction of inflammation, the remodeling of the extracellular matrix, and the de-differentiation of adult cells located near the wound^8^. Mammals have more limited capabilities than Urodels, although a transient and highly efficient regenerative process occurs, mainly triggered by progenitors cells, as reported for the digit tip and the closing of an ear hole punch and for the annual regrowth of deer antlers^9^.

Regenerative medicine aims at recovering the functionality of impaired tissues and organs through the use of various biological and tissue-engineered implants. Unfortunately, surgical implantation of both synthetic and biological materials may trigger a physiological host reaction, called foreign body reaction (FBR)^10^. Depending on the nature of the material (geometry, topography, and chemical and physical composition) or due to the host health conditions, FBR can be associated to infection, acute/chronic inflammation, and fibrous capsule formation^6,11^, negatively influencing the outcome of an implant^12^.

Mϕ play a pivotal role during FBR as well as in blastema formation, as they orchestrate all the steps by secreting biochemical mediators (chemokines, cytokines, and growth factors). There are different hypothesis to explain the mechanisms by which Mϕ react to different implanted materials^13–15^. The more established mechanism involves the complement receptors in a simil-opsonisation process triggered by host protein adsorbed on the implanted material^16,17^. Mϕ complement receptors interact with either adsorbed proteins or immunoglobulins (IgG and IgM) in order to activate the opsonization process^18^. However, recent works describe a similar Mϕ reaction and polarization, both *in vivo* and *in vitro* ^19^, where the process of opsonization and protein adsorption do not happen. Despite the Mϕ activation mechanism, there are many ways in which these events can be altered to boost the healing process reducing or eliminate fibrous tissue formation^20^.

We hypothesized that the host reaction to an implant can be controlled through the early recruitment of Mϕ into the scaffold and their *in situ* retention, along with the continuous induction of anti-inflammatory cytokines, leading to the formation of a pro-regenerative environment. In this study, we describe a biomimetic collagen scaffold functionalized with an anti-inflammatory macromolecule, chondroitin sulfate, to trigger a selected inflammatory environment that allows for the formation of a pro-regenerative environment. By modulating the response of immune cells we were able to induce the timely cascade of cellular and molecular events responsible for a functionally regenerative outcome.

## Results and Discussion

### Scaffold fabrication and implantation

**Figure 1:**
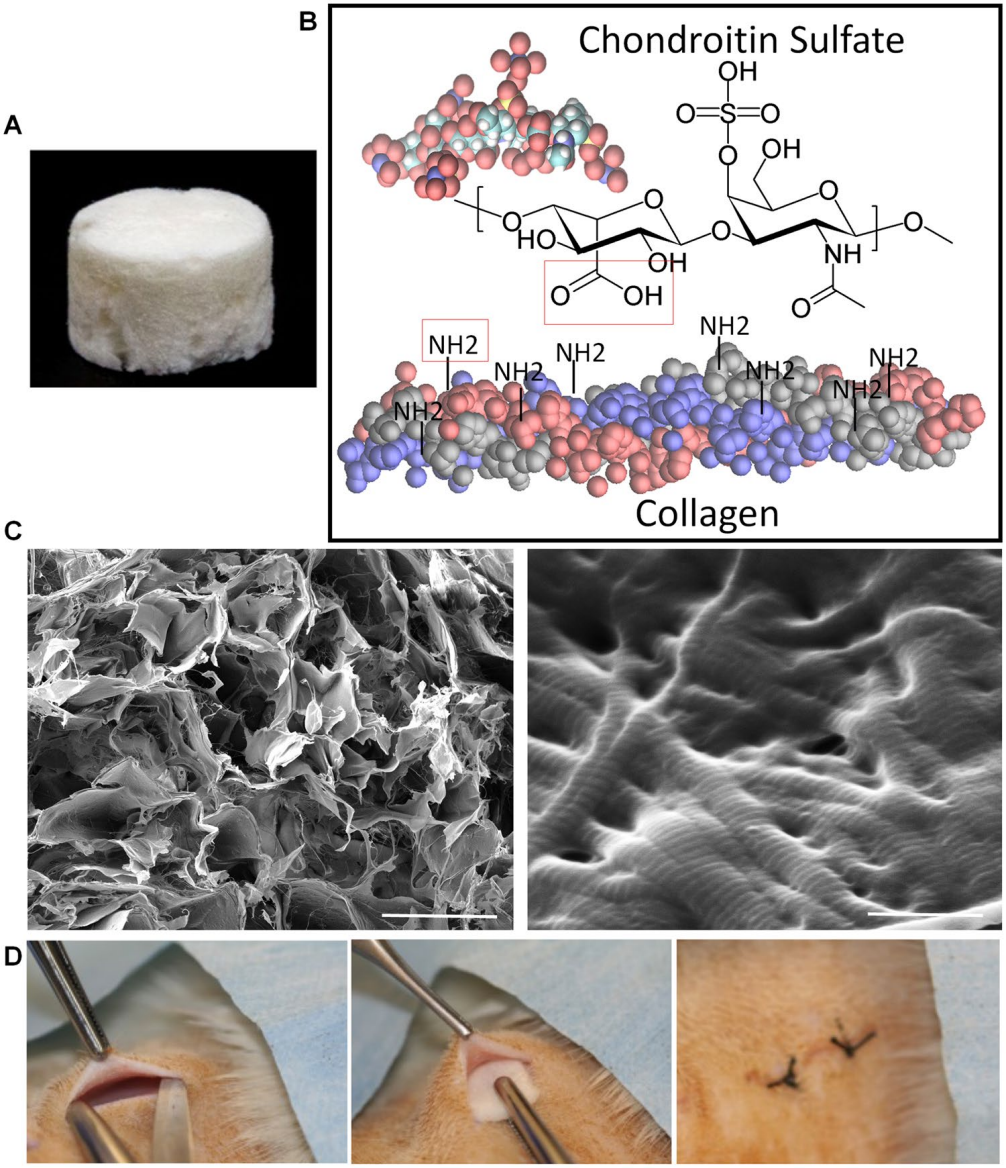
(**A**) Photograph of the collagen (CL)-chondroitin sulfate modified scaffold (CSCL). (**B**) Carbodiimide chemistry schematic to covalent link the chondroitin sulfate to Collagen structure. The carboxylic acid presented on CS sequence forms an amide bond with the free primary amines present on the collagen sequence. (**C**) SEM images showing scaffold’s porosity (on the left site) and the intact nanostructure of the collagen fibers (on the right site). (Scale bars: 100 μm and 500 nm). (**D**) Pictures showing the subcutaneous implant procedure.

A micro-porous collagen (CL)-chondroitin sulfate (CS) modified scaffold (CSCL, Fig. 1A) has been fabricated by freeze-drying technique and functionalized with chondroitin sulfate through carbodiimide chemistry (Fig. 1B). Scaffolds have been characterized by highly interconnected porosity and structured collagen fibers (Fig. 1C). CS is a glycosaminoglycan mainly present in the extracellular matrix of cartilage and in the central nervous system, where it acts as a modulator of the synaptic plasticity^21^. In mammals its expression in response to an insult activates a protective mechanism that limits the spreading of the damage to surrounding tissues^22^. CS can bind and spatially localize growth factors and ultimately exerts a strong anti-inflammatory potential^23^. We previously demonstrated that the grafting of chondroitin sulfate moieties on the surface of the CSCL was sufficient to recapitulate the ECM of the cartilage tissue^15^, supported the immune-suppressive potential of bone marrow derived mesenchymal stem cells, and modulated macrophage phenotype, both *in vitro* and *in vivo* ^24^. Based on these evidences, we hypothesized that CS could activate an alternative molecular and cellular machinery able to solve inflammation within a shorter timeframe and to create a regeneration permissive environment. To test this hypothesis, we implanted the CSCL scaffold in immune competent rats (Fig. 1D), and monitored tissue response and molecular and cellular inflammatory biomarkers at different time points (1, 3 and 7 days) until the integration of the implant within surrounding tissues (3 weeks). The time points were chosen to follow the inflammatory process, marked by the influx of polymorphonuclear leukocytes normally replaced by mononuclear Mϕ at day 1, which subsides within 3–7 days towards a constructive tissue reorganization^15^.

### Cells infiltration and transitional ECM deposition

**Figure 2:**
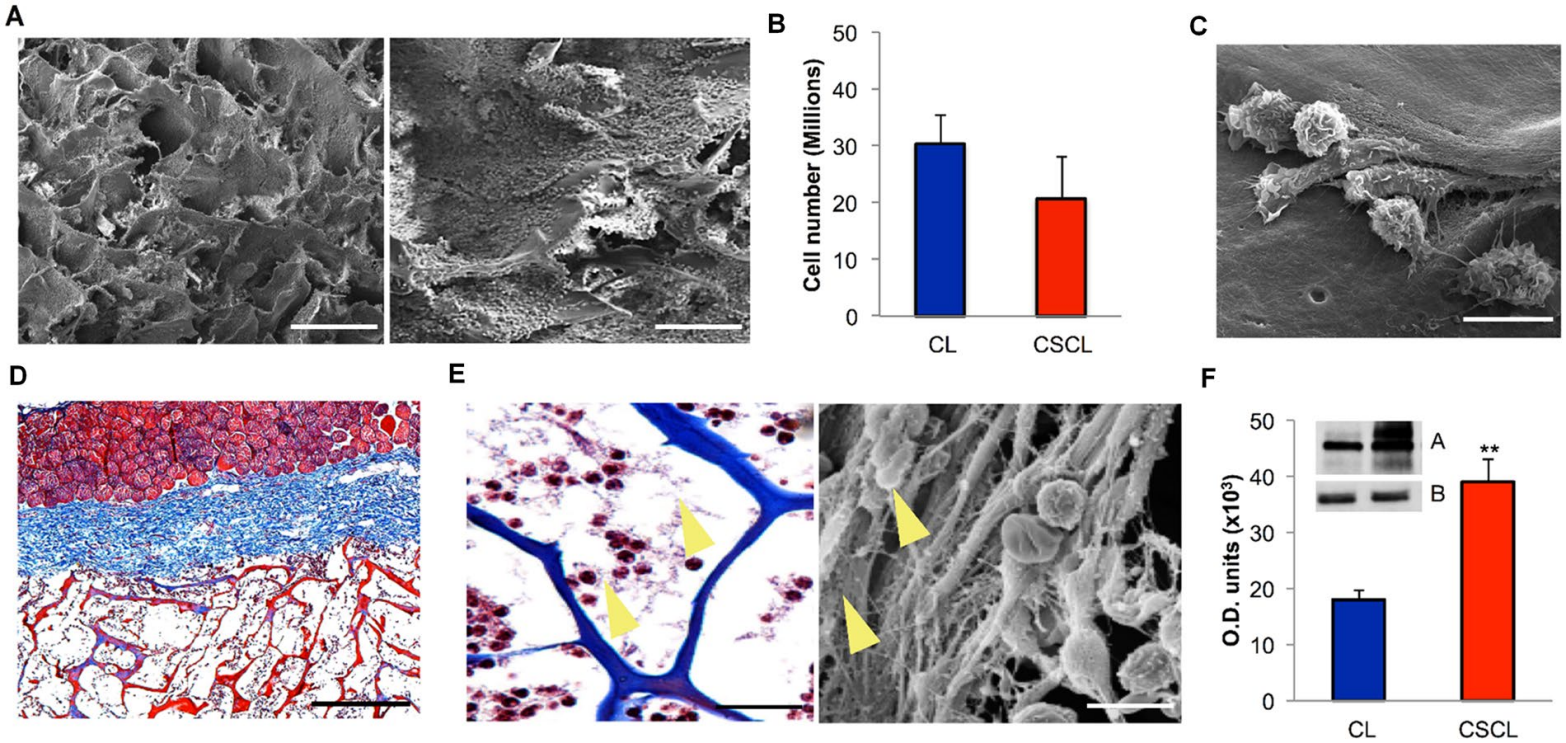
Infiltrating cells after 1 day from implantation in CSCL scaffold. (**A**) Representative SEM images showing the CSCL scaffold’ surface completely covered by cells in two different magnifications (scale bars: 200 μm and 100 μm). (**B**) Total number of cells recovered by CSCL and CL counted by flow cytometry. Graph represents mean values ± SD (n = 3). (**C**) SEM magnification to evaluate the infiltrating cells morphology on CSCL (scale bar: 10 μm). (**D**) Masson’s stained section revealed a massive infiltration of cells through the entire CSCL’ thickness coming from the surrounding vasculature (inset). (Scale bar: 200 μm). (**E**) Magnification Masson’s stained section (on the left) and a SEM image (on the right) highlighted a high level of fibronectin on the CSCL surface (yellow arrows) (scale bars: 40 μm and 15 μm, respectively). (**F**) Evaluation of fibronectin level of expression was performed on protein extracts from CL and CSCL scaffolds. Densitometric analysis, y-axis shows the optical density of protein expression (A) normalized against the control (B, GAPDH). Results are shown as means of three replicates ± SD. ** p ≤ 0.001.

One day after implantation CSCL (Fig. 2) and CL (Figure S1) were entirely colonized by a dense layer of infiltrating cells (Fig. 2A and Figure S1A), not significantly different in number evaluated by flow cytometry (Fig. 2B), with various morphologies (Fig. 2C). Histology sections suggested that infiltrating cells came from the adjacent vasculature (Fig. 2D – inset and S1B) and actively started to deposit fibrous provisional matrix **(**Fig. 2E - yellow arrows and Figure S1C). Significantly higher levels of fibronectin were detected in CSCL in comparison to unmodified collagen scaffolds (CL) at 1 day post-implant (Fig. 2F and S1D, S1E, SIF), which is required for the early creation of a regeneration permissive environment at the implant site^8^. In fact, the natural response to any implanted material involves the initial deposition of fibronectin, a ubiquitous ECM proteins that is assembled into a fibrillary network after trauma and it has been reported to be essential to facilitate cell adhesion to biomaterial surfaces^15^ and drive scar-free repair^25,26^.

### Selective gene expression of infiltrated cells

**Figure 3:**
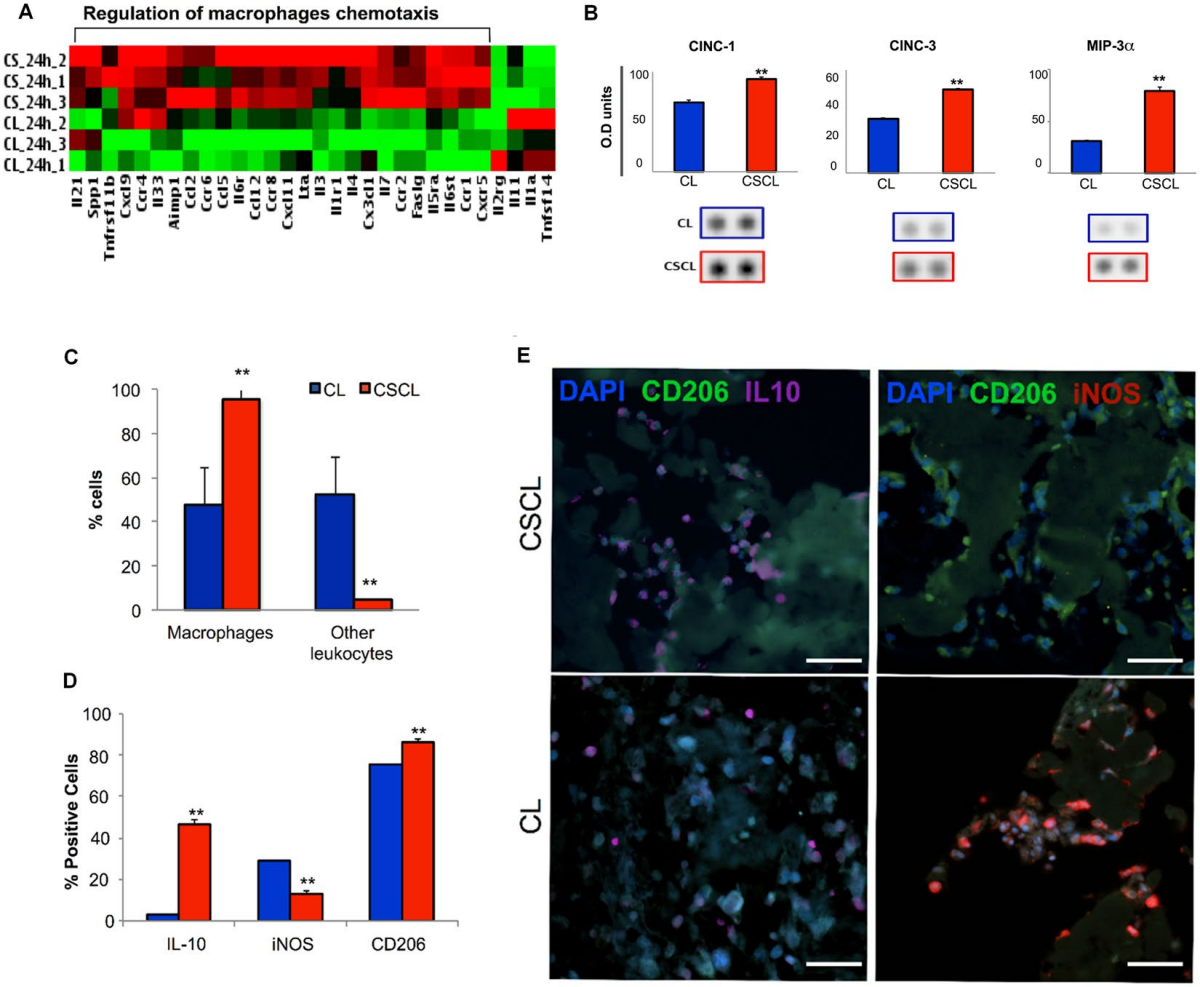
Characterization of infiltrating cells at day 1 post implant. (**A**) Heatmap of differentially expressed genes (DE) between CSCL and CL in *in vivo* explants from inflammatory cytokines and receptors PCR array. Expression levels of DE genes are displayed as color-coded: red represents over expression while green under-expression. Gene ontology analysis on over-expressed genes in CSCL shows that among the statistically significant pathways involving our data set of proteins, we found “regulation of macrophages chemotaxis” (p-value: 5.9E-18). (**B**) Rat cytokines/chemokines profiling of proteins adsorbed onto CL and CSCL scaffolds 1d after implant. Densitometric analysis. Results are shown as mean of three replicates ± SD. **p ≤ 0.001. CINC-1, CINC-3 and MIP-3α revealed different levels of abundance. (**C**) Percentage of macrophages (anti-macrophages +/anti-CD45 + cells) and other leukocytes (CD45 + cells) isolated from explants and assessed by flow cytometry. Graph represents mean values ± SD (n = 3). (**D**) Quantification of immunofluorescence staining for IL-10, iNOS and CD206 positive cells on consecutive sections Graph represents mean values ± SD (n = 10). (**E**) Representative immunofluorescence stained consecutive sections with anti-IL-10 (purple) and anti-CD206 (green) and anti-iNOS (red). The images show presence of macrophages IL-10+ (purple) within the scaffold 1-day post implant (scale bars: 50 μm).

Although the number of cells recovered from both scaffolds was not significantly different in number, the genetic profile of cells harvested from the explants showed remarkable differences between CSCL and CL (Fig. 3A). The gene ontology analysis revealed that about 50% of the 26 genes analyzed were differentially expressed in CSCL compared to CL (Table 1). Gene ontology analysis showed that up-regulated genes in CSCL were associated with regulation of Mϕ chemotaxis (i.e. *Ccl2, Ccl5*, *Ccr1, Ccr2, Ccr4, Ccr6, Ccr8, Cx3cl1, Cxcl9, Il6ra, Cxcl11, Il-4*) (p-value: 5.9E-18), while down-regulated genes were associated with a reduced inflammatory state. The analysis of the protein profile revealed a rapid induction of myeloid chemotactic chemokines (CINC-1, CINC-3, and MIP-3a) in presence of CSCL compared to CL (Fig. 3B), which was also reported by Godwin J. W. *et al*. and described as the most distinctive feature in the early phases of salamander’s limb regeneration^8^. Interestingly, in mammalian models of limb amputation, the massive recruitment of anti-inflammatory Mϕ and early tuning of the immune microenvironment has been also shown to be responsible for the formation of a transient stage, represented as the interface between two distinct events, the adult wound healing response and developmental processes^9,27^. Flow cytometric analysis of cells from the scaffolds demonstrated that 1-day post implantation Mϕ were the most represented population (95%) throughout the CSCL scaffold, whereas a mixed cell population was observed in CL (Fig. 3C). Mϕ can exhibit a pro- and anti-inflammatory phenotype depending on the local tissue environment^28^. In the classic model of inflammation after injury, the accumulation of Mϕ has been reported somewhat later, with a peak between day 3 to 7, and a progressive and significant decline by day 10 to 14^28^. Qualitative and quantitative (Fig. 3D,E) analysis showed that the Mϕ population infiltrating CSCL was predominantly associated to anti-inflammatory phenotype (IL-10^+^/CD206^+^ Mϕ). A significant reduction in the percentage of Mϕ expressing the pro-inflammatory marker *iNOS* was also observed in CSCL compared to CL.

**Table 1:** Over- and under-expressed genes in 24 h CSCL *in vivo* implant compared with CL profiled on pro-inflammatory cytokines and receptors PCR array.

Symbol	Refseq	Description	Fold Change	p-value
**Over-expressed genes in CSCL**				
Aimp1	NM_053757	Aminoacyl tRNA synthetase complex-interacting multifunctional protein 1	2,0423	0,034437
Ccl12	NM_001105822	Chemokine (C-C motif) ligand 12	3,5857	0,013407
Ccl2	NM_031530	Chemokine (C-C motif) ligand 2	2,6097	0,053334
Ccl5	NM_031116	Chemokine (C-C motif) ligand 5	2,1609	0,07928
Ccr1	NM_020542	Chemokine (C-C motif) receptor 1	2,5869	0,001323
Ccr2	NM_021866	Chemokine (C-C motif) receptor 2	3,147	0,008204
Ccr4	NM_133532	Chemokine (C-C motif) receptor 4	2,1166	0,253888
Ccr6	NM_001013145	Chemokine (C-C motif) receptor 6	3,2838	0,050775
Ccr8	XM_236704	Chemokine (C-C motif) receptor 8	3,4874	0,005749
Cx3cl1	NM_134455	Chemokine (C-X3-C motif) ligand 1	2,0435	0,011638
Cxcl11	NM_182952	Chemokine (C-X-C motif) ligand 11	2,406	0,027316
Cxcl9	NM_145672	Chemokine (C-X-C motif) ligand 9	2,0105	0,06048
Cxcr5	NM_053303	Chemokine (C-X-C motif) receptor 5	3,0484	0,001469
Faslg	NM_012908	Fas ligand (TNF superfamily, member 6)	2,9559	0,002851
Il1r1	NM_013123	Interleukin 1 receptor, type I	3,3212	0,034378
Il21	NM_001108943	Interleukin 21	2,5714	0,130204
Il3	NM_031513	Interleukin 3	3,4003	0,028243
Il33	NM_001014166	Interleukin 33	2,2283	0,205627
Il4	NM_201270	Interleukin 4	2,4614	0,046653
Il5ra	NM_053645	Interleukin 5 receptor, alpha	2,3901	0,009556
Il6r	NM_017020	Interleukin 6 receptor	2,478	0,004808
Il6st	NM_001008725	Interleukin 6 signal transducer	2,2308	0,001629
Il7	NM_013110	Interleukin 7	3,5534	0,000298
Lta	NM_080769	Lymphotoxin alpha (TNF superfamily, member 1)	2,9841	0,025761
Spp1	NM_012881	Secreted phosphoprotein 1	2,3364	0,109091
Tnfrsf11b	NM_012870	Tumor necrosis factor receptor superfamily, member 11b	3,33	0,172409
**Under-expressed genes in CSCL**				
Il11	NM_133519	Interleukin 11	0,2471	0,226938
Il1a	NM_017019	Interleukin 1 alpha	0,1181	0,009542
Il2rg	NM_080889	Interleukin 2 receptor, gamma	0,3161	0,349324
Tnfsf14	NM_001191803	Tumor necrosis factor (ligand) superfamily, member 14	0,1531	0,011446

### Downstream effects of differential cells’ recruitment

We next evaluated the downstream effect of the environment produced by the early recruitment of IL-10^+^/CD206^+^ Mϕ by CSCL, analyzing CSCL explants at 3 and 7 days.

**Figure 4:**
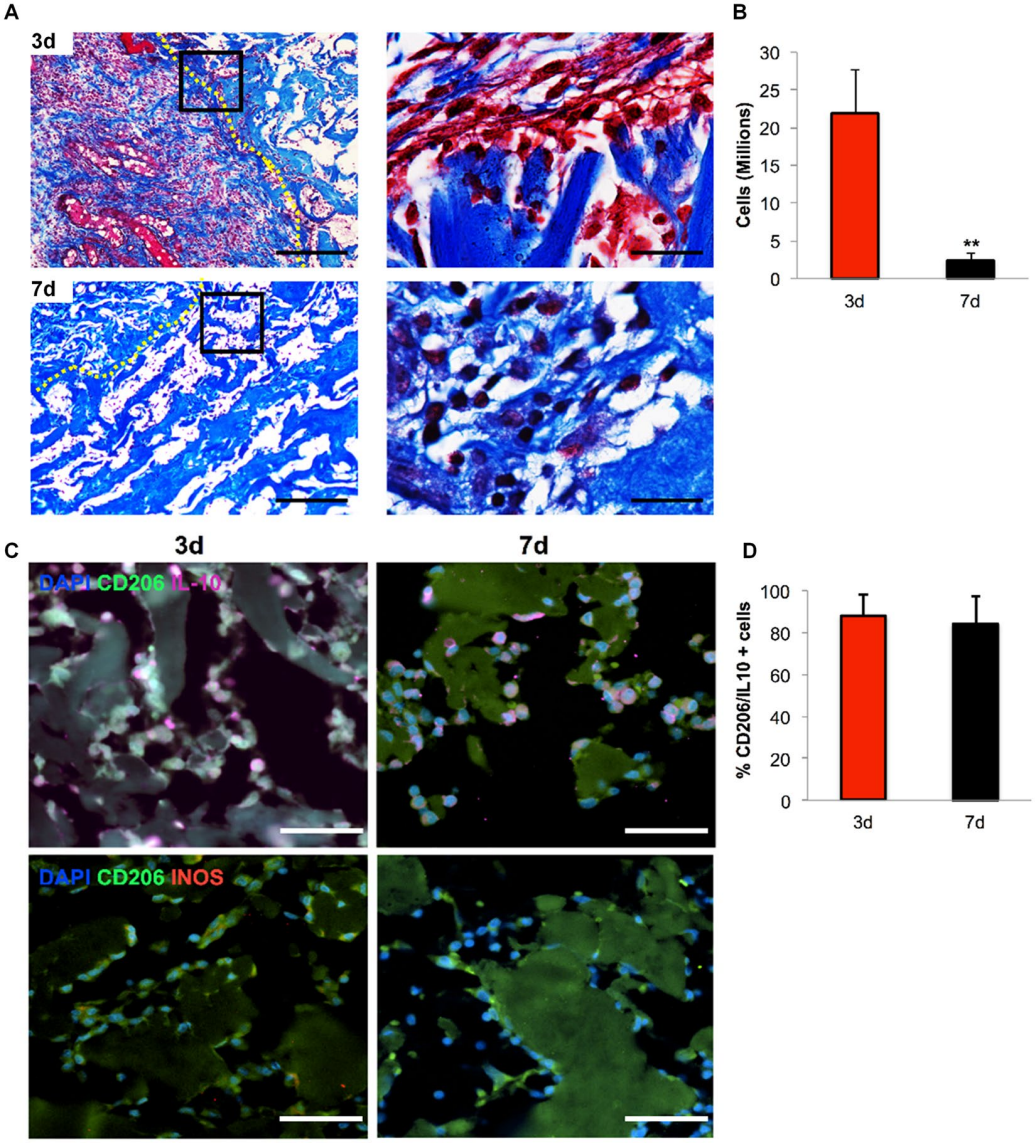
Switching off of inflammation. (**A**) Representative images of Masson’s stained sections of CSCL (dotted yellow lines mark the interface with the native tissue) at 3 (top) and 7 days (bottom) (Scale bars: 100 μm, 50 μm). Images highlight dampen of the inflammation representing in a decrease of infiltrating cells from the surrounding tissue and an augment of extracellular matrix deposition. (**B**) Reduction in the number of cells harvested from explanted CSCL at 3 and 7 days. Values are mean ± SD (n = 3) (n = 3, **p < 0.01). (**C**) Representative immunofluorescence consecutive sections showing anti (IL-10)- and pro(iNOS)- inflammatory markers. Cells are counterstained with anti-Macrophages antibody. A progressive reduction of IL-10+ and iNos+ macrophages between 3 and 7 days is shown (scale bars 50 μm). (**D**) Flow cytometric analysis shows the percentage of IL10+/CD206+ macrophages at 3 and 7 days from CSCL implant.

The total number of cells harvested from the CSCL was reduced overtime (Fig. 4A **)** and correlated to the presence of fibronectin matrix observed at the interface with the scaffold, together with the augmentation of collagen deposition (Fig. 4A, magnifications). Further analysis confirmed qualitatively (Fig. 4A) and quantitatively (Fig. 4B) these observations, revealing that although the cell number was significantly decreased at day 7, a persistent presence of Mϕ displaying the anti-inflammatory phenotype (IL-10^+^/CD206^+^ Mϕ) was observed between 3 and 7 days post implant (Fig. 4D). Consistently with the activation of specific chemotaxis-associated pathways^29^, only 5% of the cells were positively stained for the pro-inflammatory marker iNOS, as revealed by flow cytometry (Fig. 4C).

**Figure 5:**
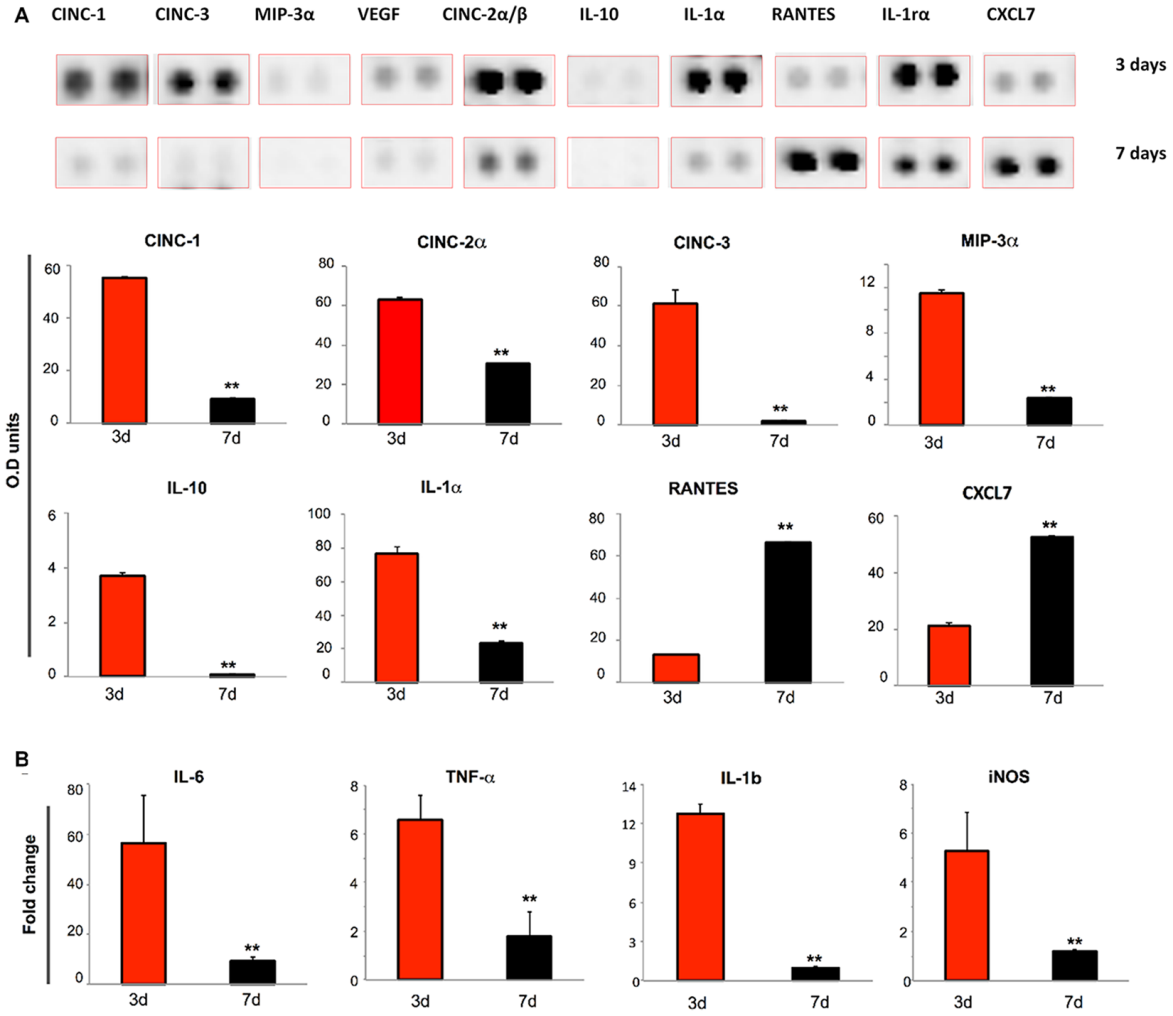
Inflammatory proteins expression induced by CSCL. (**A**) Differences in the rat cytokines/chemokines profiling of CSCL at 3 days and 7 days. Proteins adsorbed on CSCL surface were extracted. Proteins with different levels of abundance at 3 and 7 days are represented. Bar graph presents mean densitometry units of each spot. Values are presented as the mean ± SD. (**B**) Quantitative PCR analysis for the pro-inflammatory (*Il-6, Tnf-α Il-1β, iNos)-* associated markers at 3 and 7 days from CSCL implants. Expression levels normalized to the reference gene (*Gapdh*). Data are represented as fold-change compared with expression observed in subcutaneous tissues explanted from rats in absence of inflammation. Values are mean ± SD (n = 3). Asterisks depict significant differences between 3 and 7 days (**p < 0.01).

Moreover, at day 3, the levels of chemoattractant chemokines (CINC-1, CINC-3, CINC2α/β, MIP3α) analyzed by proteomic array were still significantly higher than the control (Figure S2), but markedly decreased by day 7 (Fig. 5A). We hypothesized such reduction to be correlated to a potential resorption of the fibronectin network in CSCL scaffolds as compared to the control (CL) (Figure S2). To test this correlation, we evaluated the levels of fibronectin at day 7 and we found that the CL scaffold was still filled by a fibronectin matrix, which surrounded population of cells that were still infiltrating the entirety of the scaffold. On the contrary, in CSCL the fibronectin was replaced by the deposition of stable ECM as highlighted by broad blue area in the histological images. We then elucidated the role of the early infiltration of anti-inflammatory Mϕ, into the scaffold, their retention *in situ*, and the simultaneous release of anti-inflammatory cytokines as part of a distinct regenerative program activated by the CSCL scaffold. To understand how the persistent presence of anti-inflammatory Mϕ could influence the inflammatory status, quantitative PCR was performed on the cells isolated from the scaffolds 3 and 7 days post implantation and showed a significant (p < 0.01) down-regulation of markers associated with pro-inflammatory events (*Il-6, Il-β, Tnf-α* and *iNos*, Fig. 5B). Taken together these findings suggest that the immune-modulatory role of CSCL, allowed the activation of a differential immune response that allowed the early recruitment and retention of anti-inflammatory Mϕ, which in turn led to the resolution of the acute inflammatory phase following surgical implantation.

### Scaffold remodeling and integration

**Figure 6:**
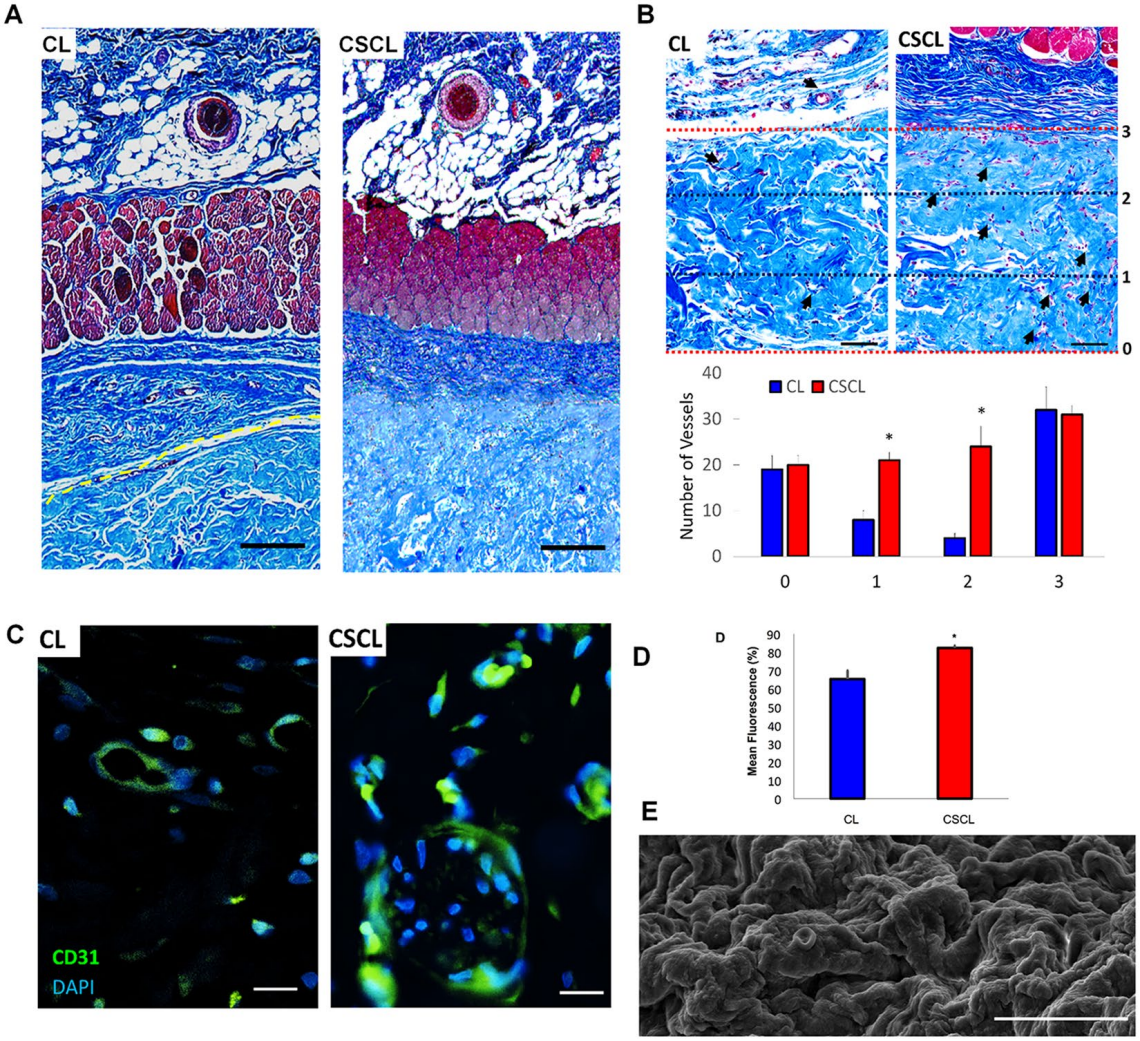
Scaffold integration. (**A**) Representative Masson’s trichromic stained whole section shows the different integration of the scaffold along all its cross-section between CL and CSCL (1 mm). (**B**) Representative Masson’s trichromic stained whole section shows the complete integration of the scaffold along all its cross-section (Scale bars: 100 μm). Arrows indicate the vessels inside the scaffold. The graph indicates the presence of the vessels throughout the scaffolds’ thickness (distributed in the 3 areas) (n = 3, *p < 0.05). (**C**) Representative CD31 immunofluorescence stained section used for quantification (scale bars: 20 μm). (**D**) Quantification of CD31 immunofluorescence stained section (n = 3, *p < 0.05). (**E**) SEM image of the scaffold surface shows completely remodeling of the scaffold (Scale bar: 10 μm).

We also evaluated whether the regeneration-permissive environment created by our biomimetic scaffold had a long-term effect in terms of blood vessel morphogenesis, collagen fibril organization and the scaffold’s integration in the surrounding tissue. We isolated the areas within and surrounding the scaffold 21 days post implant and analyzed the *de novo* deposition and organization of the extracellular matrix^30,31^. The histomorphometric analysis showed complete integration of the scaffold within the tissue with a 100% histomorphometric index^32^ (Fig. 6A). The scaffold integration into the native tissue was also suggested by an area of highly vascularized connective tissue (Fig. 6B), which was beneficial to increase blood supply and is required to permit exchange of oxygen and nutrients between the implant and the body^33^. The histological analysis of the CSCL 21 days after implant showed increased new vessels formation compared to CL. Also, the immunohistochemical staining of CD31 (PECAM1) revealed an increase of positive cells in the tissue sections obtained from CSCL **(**Fig. 6C,D **)**, suggesting the successful integration of the scaffold within the surrounding tissue^3^. The majority of the CD31^+^ cells was associated with histologically mature vascular structures, distributed across the entire scaffold thickness **(**Fig. 6B **)**, and was accompanied by a thorough remodeling of the surface of the scaffold (Fig. 6E), and at the interface with the surrounding native tissue.

We propose that the biomimetic properties of the CSCL triggered an early cascade of events that ultimately influenced the production of functional blood vessels, culminating in the increase of vascular density and collagen fibrils organization, as shown by immunohistochemistry and qPCR arrays. The same features were not observed in CL samples. In fact, CL scaffold was not sufficient to control the host response following the implant showing a consequent failure in the integration with the surrounding tissue (Fig. 6A).

**Figure 7:**
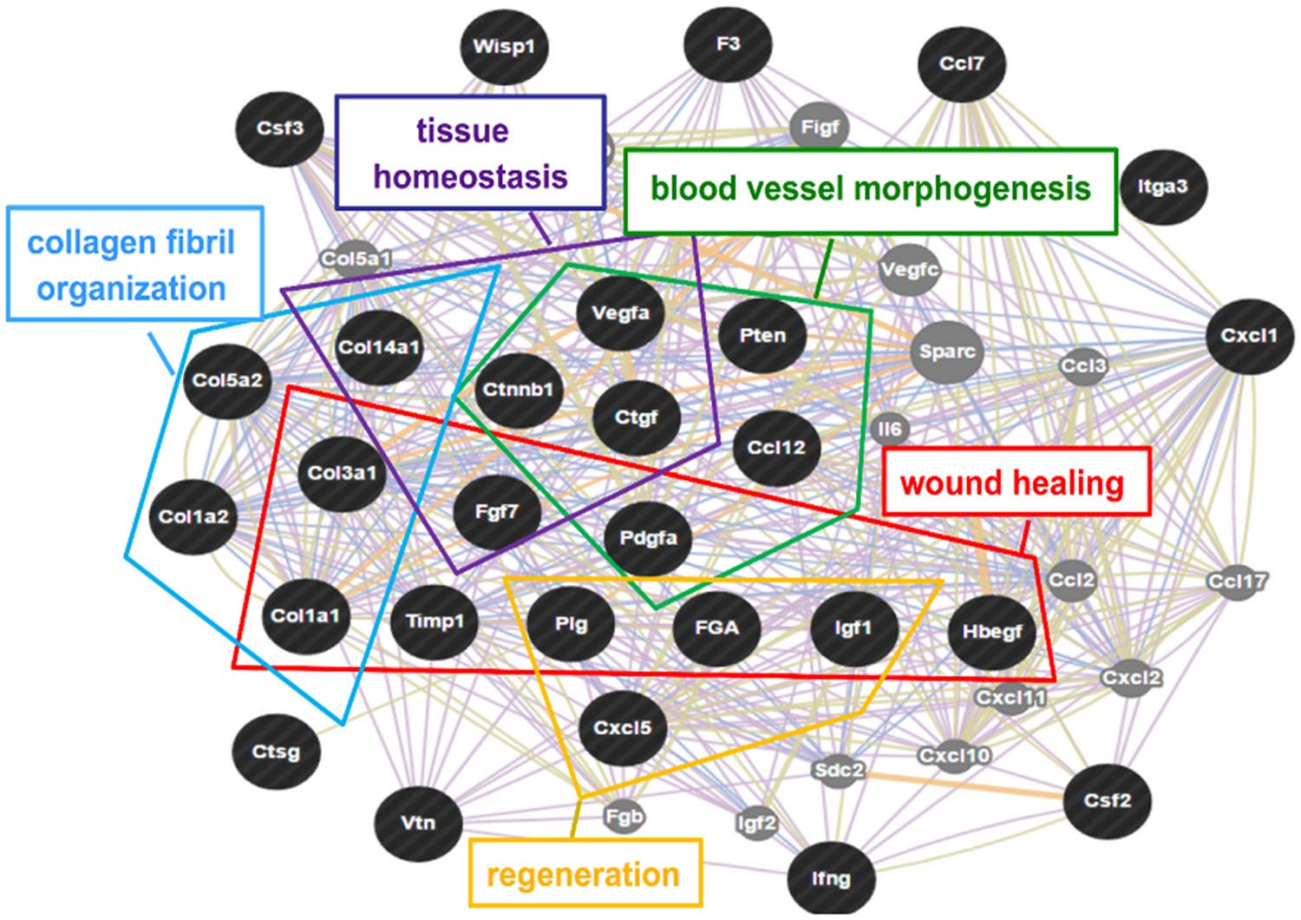
Differential genetic profile induced by CSCL at 21 days. Functional classification of over-expressed genes in CSCL *in vivo* implants from wound healing PCR array by using GENEMANIA web analysis tool.

To confirm the occurrence of neo-angiogenesis we also analyzed the presence of collagen IV, an important component of the basal lamina of mature vessels^34^. Western blot analysis demonstrated an increased presence of Collagen IV in the CSCL compared to its CL counterpart, with the concomitant marked reduction of deposited fibronectin (Figure SF3). As mentioned above, fibronectin deposition in ECM is tightly regulated during the regenerative process^35^. The decrease of the fibronectin matrix in CSCL was indicative of a reduced fibrotic scarring and suggested the successful integration of the implant^36^ (Figure SF4). To further corroborate these findings, we assessed the expression of 84 genes involved in the wound healing and regeneration process. Statistically significant changes in the expression of 28 genes were detected in CSCL compared to CL (33% variation, Table 2). A differential expression was observed in genes belonging to the biological processes involved in tissue restoration, such as blood vessel morphogenesis (*Vegfa, Pten, Ccl12, Pdgfa, Ctgf, Ctnnb1*), tissue homeostasis (*Col14a1, Ctnnb1, Ctgf, Fgf7, Vegfa*), collagen fibrils organization (*Col14a1, Col5a2, Col3a1, Col1a1, Col1a2*), and wound healing (*Col1a1, Fgf7, Igf1, Hbegf, FGA, Plg, Pdgfa, Timp1*) (Fig. 7). The observed scaffold integration and the neo-angiogenesis confirmed the progression toward the tissue-remodeling phase following the activation of anti-inflammatory molecules^11,37^.

**Table 2:** List of genes found over-expressed among the 84 tested through the wound healing PCR array in 21 d CSCL *in vivo* implant compared to CL.

Symbol	Refseq	Description	Fold Change	95% CI
Pten	NM_031606	Phosphatase and tensin homolog	6,09	(0.00001, 19.52)
Csf2	NM_053852	Colony stimulating factor 2 (granulocyte-macrophage)	5,24	(0.00001, 23.34)
Col1a1	NM_053304	Collagen, type I, alpha 1	5,22	(0.00001, 14.46)
Csf3	NM_017104	Colony stimulating factor 3 (granulocyte)	5,02	(0.00001, 23.34)
Pdgfa	NM_012801	Platelet-derived growth factor alpha polypeptide	4,93	(0.00001, 20.54)
Col1a2	NM_053356	Collagen, type I, alpha 2	4,05	(0.00001, 15.35)
Ccl7	NM_001007612	Chemokine (C-C motif) ligand 7	3,79	(0.00001, 14.17)
Hbegf	NM_012945	Heparin-binding EGF-like growth factor	3,66	(0.00001, 16.11)
Wisp1	NM_031716	WNT1 inducible signaling pathway protein 1	3,58	(0.00001, 17.13)
Ctnnb1	NM_053357	Catenin (cadherin associated protein), beta 1	3,20	(0.00001, 13.95)
Itga3	NM_001108292	Integrin, alpha 3	3,17	(0.00001, 13.52)
Plg	NM_053491	Plasminogen	3,15	(0.00001, 12.00)
Col5a2	NM_053488	Collagen, type V, alpha 2	3,11	(0.00001, 11.58)
Ctsg	NM_001106041	Cathepsin G	2,95	(0.00001, 9.59)
Cxcl1	NM_030845	Chemokine (C-X-C motif) ligand 1	2,69	(0.00001, 10.77)
Col3a1	NM_032085	Collagen, type III, alpha 1	2,61	(0.00001, 9.05)
Fga	NM_001008724	Fibrinogen alpha chain	2,53	(0.00001, 9.22)
Col14a1	NM_001130548	Collagen, type XIV, alpha 1	2,48	(0.00001, 9.11)
Vtn	NM_019156	Vitronectin	2,47	(0.00001, 8.03)
Timp1	NM_053819	TIMP metallopeptidase inhibitor 1	2,41	(0.00001, 8.42)
Ccl12	NM_001105822	Chemokine (C-C motif) ligand 12	2,36	(0.00001, 9.91)
Fgf7	NM_022182	Fibroblast growth factor 7	2,29	(0.00001, 8.14)
Igf1	NM_178866	Insulin-like growth factor 1	2,26	(0.00001, 6.91)
Cxcl5	NM_022214	Chemokine (C-X-C motif) ligand 5	2,26	(0.00001, 8.34)
Vegfa	NM_031836	Vascular endothelial growth factor A	2,25	(0.00001, 7.93)
F3	NM_013057	Coagulation factor III (thromboplastin, tissue factor)	2,17	(0.00001, 7.43)
Ifng	NM_138880	Interferon gamma	2,12	(0.00001, 6.74)
Ctgf	NM_022266	Connective tissue growth factor	2,07	(0.00001, 6.23)

**Figure 8:**
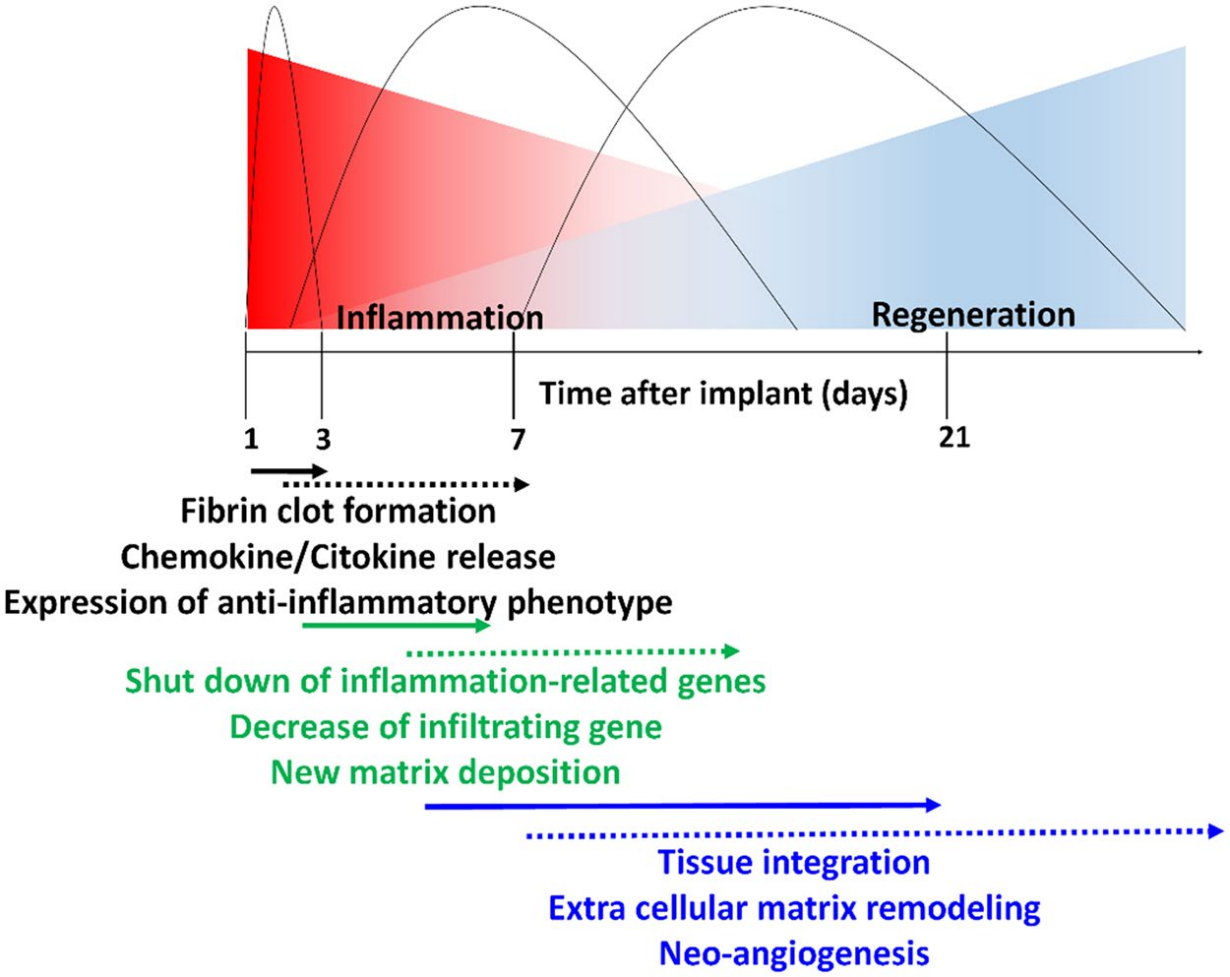
Schematic description of the regenerative events induced by the CS functionalization (hard line). The presence of CS results in the anticipated occurrence and shorter duration of the cascade of events following scaffold implantation. The dotted line shows the established wound healing phases.

This study provides a comprehensive analysis of the molecular, cellular and tissue events occurring over time at the site of implant of a biomimetic scaffold able to guide the immune response towards an anti-inflammatory environment. These events were probably triggered by the combination of chemical (scaffold composition and surface chemistry) and structural (pore size and interconnected porosity) cues that are known to favor the localization of growth factors with anti-inflammatory potential^38,39^, thus promoting cells’ infiltration and retention throughout the scaffold thickness, respectively^33^. The modification of the collagen scaffold with an immune-modulatory molecule (CS) enhanced the recruitment of Mϕ with anti-inflammatory phenotype, and reduced the infiltration of detrimental pro-inflammatory leukocytes. We demonstrated that the down-regulation of the inflammatory signaling cascade triggered by anti-inflammatory Mϕ was able to accelerate the initiation of the regenerative process^40^ and to lead to blood vessel formation, collagen fibril re-organization and scaffold integration. A schematic description of the regenerative events induced by the CS functionalization (hard line) is reported in Fig. 8. The early occurrence and shorter duration of the events activated by CSCL implantation is depicted in comparison to the well-established sequence of events occurring in the physiological wound healing process.

Our study is a proof of concept demonstration that by tuning the early events occurring at the scaffold/tissue interface, it is possible to affect the final outcome of a tissue engineering implant.

## Conclusions

This study paves the way for the design and improvement of immune modulatory tissue engineering approaches aiming at expediting the process of wound healing toward faster implant integration to achieve a functional tissue restoration. The evidences we provided suggest that the chemical and structural properties of an implantable biomimetic material are sufficient to stimulate the body toward tissue healing. The anti-inflammatory and structural features of the CSCL presented in this study indicate that this scaffold was able to recruit immune cells soon after the implant, retain them at the site, and tune their phenotype to avoiding the immunological rejection observed when CL is crosslinked with other agents and achieve complete incorporation into the tissue.

## Materials and Methods

### Scaffold preparation

Collagen (CL) type I from Bovine tendon (Nitta Casing) was dissolved at a concentration of 40 mg/mL in a solution of 0.05 M acetic acid. The pH of the solution was adjusted to 7.4 with NaOH 0.1 N. Condroitin sulphate (CS, Carbosynth) was added to the collagen solution at a molar ratio of 10:1 between CL and CS. The slurry was poured into a 24-well plate, frozen at −80 °C for 3 h and lyophilized at 20 mTorr. The scaffolds were subsequently cross-linked (CSCL) for 4 h at 37 °C using 50 mM 2-(N-morpholino)ethanesulfonic acid, 5 mM 1-Ethyl-3-(3-dimethylaminopropyl)carbodiimide (EDC), 5 mM N-Hydroxysuccinimide (NHS). Scaffolds were rinsed twice for 1 h with 0.1 M disodium phosphate, and 6 times for 24 hours with 2 M sodium chloride, and finally with distilled water to remove residual EDC. Scaffolds were air dried and sterilized by UV irradiation for 30 min under laminar flow hood and equilibrated in culture medium at 37 °C for 5 hours before being used.

### Scanning electron microscope

Scaffolds were dehydrated with ethanol solutions (30%, 50%, 75%, 85%, and 95% each for 2 hours), and placed overnight in a dryer at RT before being coated by 7 nm of Pt/Pl for examination on an FEI Nova NanoSEM 230. The average pore diameter of the scaffolds was measured from SEM images (n = 5). For each image, 20 different pores were randomly selected and their diameters were measured using Image-J software.

*Animals*. Adult Lewis rats (Charles River Laboratories. Houston, Texas. USA), were used for material implantation. The animals were divided in 4 groups (1, 3, 7 and 21 days). Animal studies were conducted following approved protocols (AUP-0115-0002) established by Houston Methodist Research Institute’s Institutional Animal Care and Use Committee (IACUC) in accordance with the guidelines of the Animal Welfare Act and the Guide for the Care and Use of Laboratory Animals. The skin incisions were made on both sides of the back of each animal (Left side: CL. Right side: CSCL) under sterile technique and isoflurane inhalation anesthesia. After accessing the subcutaneous plane, 2 cm^2^ pockets were created and the scaffolds (1 cm diameter, 0.3 cm thick) were placed in each pocket. When designated time points were reached, animals were euthanized under IACUC’s guidelines.

### Tissue isolation and samples preparation

After harvesting the materials and adjacent tissues, specimens were fixed for histological and SEM analyses, preserved either in RNAlater® solution (Ambion, Life Technologies Corp.) or lysis buffer for gene expression and proteomic assays, digested with collagenase (Life Technologies) for flow cytometric analysis.

### Histological and immunohistological analysis

Constructs were washed in PBS, fixed with 3.7% formaldehyde in PBS overnight, dehydrated through a graded series of ethanol solutions, embedded in paraffin, and sectioned at a thickness of 10 μm. For histological observation, sections were deparaffinized, rehydrated, stained with Trichrome Stain (Abcam; ab150686), and analyzed by Nikon Histological Microscope (Eclipse Ci-E/Ci-L/Ci-S). The NIS-Elements software was employed to quantify cell infiltration, collagen deposition and vascular density.

Immunofluorescence analysis was performed on adjacent 10-micron sections to assess the expression and the immunolocalization of IL-10 (Bioss bs-0698R), CD206 (Biorbyt), iNOS (Abcam ab3523), CD31 (LSBio LS-C43480) and Anti-Rat Macrophages (Macrophage Marker Monoclonal Antibody (HIS36), PE Catalog Number A18516, Invitrogen). After cooling for 4 hours at RT, the slides were rinsed in PBS for 15 minutes and blocked in 10% goat serum and 0.3% Triton X-100 (Sigma-Aldrich) for 1 hour at RT and then incubated overnight with the primary antibody at 4 °C. Subsequently, the slides were incubated with the secondary antibody for 2 hours. The iNOS, IL-10 and CD206 primary antibodies (1:100 in blocking solution) were detected by incubation for 2 hours at RT with DK Anti-Rb 555 (1:500) secondary antibody (Life Technologies), and then rinsed three times with PBS. CD31 and Anti-Rat Macrophages, directly conjugated antibodies, were incubated in the dark for 2 hours at RT after the blocking step. The air-dried slides were mounted in fluorescent mounting media containing DAPI (Prolong Gold; Invitrogen-Molecular Probes) and imaged with a Nikon Histological Microscope.

### SEM samples preparations

Three samples/animal group were evaluated by SEM. The samples were washed twice with a 0.1 M sodium cacodylate buffer for 10 min. All the samples were fixed overnight at 4 °C with glutaraldehyde 2.5%, paraformaldehyde 1% in PBS (pH 7.4). Dehydration was achieved using a graded series of ethanol solutions (25%, 50%, 70%, 90% and 100% for 10 min/each). Specimens were mounted on metal stubs and stored in a vacuum desiccator for 48 h. In order to perform the SEM analysis (FEI Quanta 400 ESEM FEG), the samples were sputter coated with 7 nm of Pt/Pd with Plasma Sciences CrC-150 Sputtering System (Torr International, Inc) and imaged at 10 kV.

### Scaffold handling and western blotting analysis

The proteins adsorbed on the scaffolds were collected as follows: the scaffolds were cut in small pieces and then resuspended in cold RIPA buffer (Thermo Fisher Scientific, Waltham, MA) supplemented with protease and phosphatase inhibitors. Proteins were extracted by sonication of the samples and then quantified by the Bradford assay (Bio-Rad, Hercules, CA). For each sample, 40 μg of proteins were separated through a 4–15% polyacrylamide gel for 1 hour 30 min at 120 V. Proteins were transferred on PVDF membranes, blocked for 2 hour in 5% non-fat milk and incubated overnight at 4 °C with anti-Fibronectin (Abcam; 1:1000), and anti-Collagen-IV (Abcam; 1:1000) primary antibodies. The membranes were then incubated with HRP (horseradish peroxidase)-conjugated anti-Rabbit IgG and anti-Mouse IgG (Sigma-Aldrich) secondary antibodies for 1 hour. The bands were detected by chemiluminescence using the SuperSignal West Dura Chemiluminescent Substrate (Thermo); images were visualized and acquired with ChemiDoc XRS+ System and Image Lab software (Bio-Rad).

### Cytokine and chemokine proteome profiling

The Rat Cytokine array panel A (R&D system, Minneapolis, MN) was employed for the profiling of the chemokines and cytokines secreted upon CL and CSCL scaffolds implantation. This array allows the detection of 29 different molecules. We collected and quantified the proteins adsorbed on the scaffolds using the procedure described above (Bio-Rad). The results were obtained using the manufacturer’s instruction. The protein array images were scanned and pixel density analyzed by Molecular Imager ChemiDoc XRS System + Image Lab Software v.4.1 (BioRad). The experiments were performed in triplicate.

### Flow cytometry

Flow cytometric analysis was performed on immune cells infiltrating into the scaffolds at 1, 3 and 7 days from implant. To harvest cells scaffolds were digested with collagenase type I (2 mg/ml prepared in Hank’s Balanced Salt Solution with calcium and magnesium, Life Technologies) for 30 min at 37 °C. Cell suspensions were filtered through 70 µm nylon mesh (BD Biosciences) to remove cell clumps and scaffold debris, spun at 500 g for 5 min, and fixed with 70% EtOH. After fixation, cells were washed with FACS buffer (BSA 0.1%). Cells were labeled with anti-CD45 (BioLegend), anti-macrophages (eBiosciences) anti-CD206 (Biorbyt). Intracellular staining for IL-10 (Bioss) was performed using a commercial kit for cellular fixation and permeabilization (BD Biosciences). All analyses were based on control cells incubated with isotype-specific IgGs or IgM to establish background signal. A minimum of 20,000 events per sample was analyzed using a BD LSR Fortessa™ cell analyzer (BD Biosciences, San Jose, CA). Data analysis was performed by FlowJo (Tree Star, Ashland Inc., O).

### RT2 Profiler PCR Array and Bioinformatic analysis

Total RNA was extracted from explanted CL and CSCL scaffolds using Trizol reagent (Invitrogen), and purified to eliminate genomic DNA, protein and organic contaminations using the RNeasy Mini Kit (Qiagen). The concentration and integrity of all RNA samples were assessed using the NanoDrop ND-2000 spectrophotometer (NanoDrop Technologies). cDNA synthesis was performed using the RT2 First Strand Kit (Qiagen) according to the manufacturer’s instructions. Rat Inflammatory Cytokines & Receptors and Wound Healing RT2 Profiler PCR Arrays were used to analyze the scaffolds explanted at 1 and 21 days, respectively. Plates were subjected to real-time PCR with a two-step cycling program in an ABI 7500 Fast Sequence Detection System (Applied Biosystems) using SYBR green RT2 qPCR Master Mix (Qiagen). The resulting threshold cycle values were analyzed through the SABiosciences Web-based PCR Array Data Analysis Software version 3.5 (http://www.sabiosciences.com/pcr/arrayanalysis.php).

Gene ontology (biological processes) was analyzed for over-expressed genes between CL and CSCL in 1-day explants by using NIH DAVID web tool (http://david.abcc.ncifcrf.gov), followed by REVIGO (http://revigo.irb.hr) to visualize the top enriched GO terms (biological processes). The genes whose expression was found over-expressed in CSCL compared with CL in 21 days were functionally annotated and clustered by applying GENMANIA web tool (http://www.genemania.org). The overall connectivity of the identified proteins was determined using the functional protein association network tool available on STRING (http://string-db.org/). STRING was also employed as a search tool for the Retrieval of Interacting Genes/Proteins.

### Quantitative real-time PCR

At 3 and 7 days explants were processed as reported above to extract RNA and synthesize cDNA. Transcribed products were analyzed using commercially available master mix and the appropriate target probes (IL-6: Rn01410330_m1, IL-1β: Rn00580432_m1, iNOS: Rn00561646_m1, TNF-α: Rn01525859_g1) on an ABI 7500 Fast Sequence Detection System (Applied Biosystems, Foster City, CA).

### Statistical methods

Statistical analysis was performed using GraphPad Instat 3.00 for Windows (GraphPad Software, La Jolla, CA, USA). Three replicates for each experiment were performed and the results were reported as mean ± standard deviation. A p value ≤ 0.05 was considered as significant, p > 0.01 highly significant. One-way ANOVA analysis was used for multiple comparisons through the Student-Newman-Keuls test.

## Electronic supplementary material

Supporting Information
