# Supplementary material for: Immune tuning scaffold for the local induction of a pro-regenerative environment
Source: Sci Rep. 2017 Dec 5;7:17030. doi: 10.1038/s41598-017-16895-0 (PMC5717048; doi:10.1038/s41598-017-16895-0)
Supplement: Supplementary file 1 — Supporting Information [file 41598_2017_16895_MOESM1_ESM.pdf]

**Supplementary information**

# Immune tuning scaffold for the local induction of a pro-regenerative environment.

*Bruna Corradetti[1,2#], Francesca Taraballi[3,4#], Claudia Corbo[3,5] , Fernando Cabrera[3], Laura Pandolfi[3], Silvia Minardi[3], Xin Wang[3], Jeffrey Van Eps[3], Guillermo Bauza[3,4,6], Bradley Weiner[4] and Ennio Tasciotti[3,4,6*].*

[1]Department of Nanomedicine, Houston Methodist Research Institute, 6670 Bertner Ave., Houston, TX 77030, USA.

[2]Department of Life and Environmental Sciences, Polytechnic University of Marche, via Brecce Bianche, 60131, Ancona, Italy.

[3]Center for Biomimetic Medicine, Houston Methodist Research Institute, 6670 Bertner Ave., Houston, TX 77030, USA

[4]Department of Orthopedics & Sports Medicine, Houston Methodist Hospital, 6550 Fannin St., Houston, TX, 77030, USA

[5]Center for Nanomedicine, Brigham and Women's Hospital, Harvard Medical School, Boston, MA

[6]Center for NanoHealth, Swansea University Medical School, Swansea University Bay, Singleton Park, SA2 8PP, Wales, UK.

#equal contribution

*etasciotti@houstonmethodist.org

## Quantification of fibronectin via ELISA assay

The amount of fibronectin deposited on CL and CSCL scaffold after 24hs have been further quantified by enzyme-linked immunosorbent assay (ELISA) (Abcam). Proteins were extracted from 3 independent scaffold by sonication and loaded after a dilution 1:10000 in technical triplicate. The assay has been performed following the instructions of the manufacturer.

## Quantification of fibronectin by immunohistochemistry

Immunofluorescence analysis was performed on adjacent 10-micron sections to assess the expression and the immunolocalization of Fibronectin (Abcam ab199056). A total of 3 slides per animal has been

stained. After the antigen retrieval treatment (TRIS-EDTA, pH9) the slides were rinsed in PBS-T for 15 minutes and blocked in 10% goat serum and 0.025% Triton X-100 (Sigma-Aldrich) for 1 hour at RT and then incubated overnight with the primary antibody at 4°C (1:250). Subsequently, the slides were incubated with the secondary antibody for 2 hours. DK Anti-Rb 555 (1:500) secondary antibody (Life Technologies), and then rinsed three times with PBS. The air-dried slides were mounted in fluorescent mounting media containing DAPI (Prolong Gold; Invitrogen–Molecular Probes) and imaged with a Nikon Histological Microscope

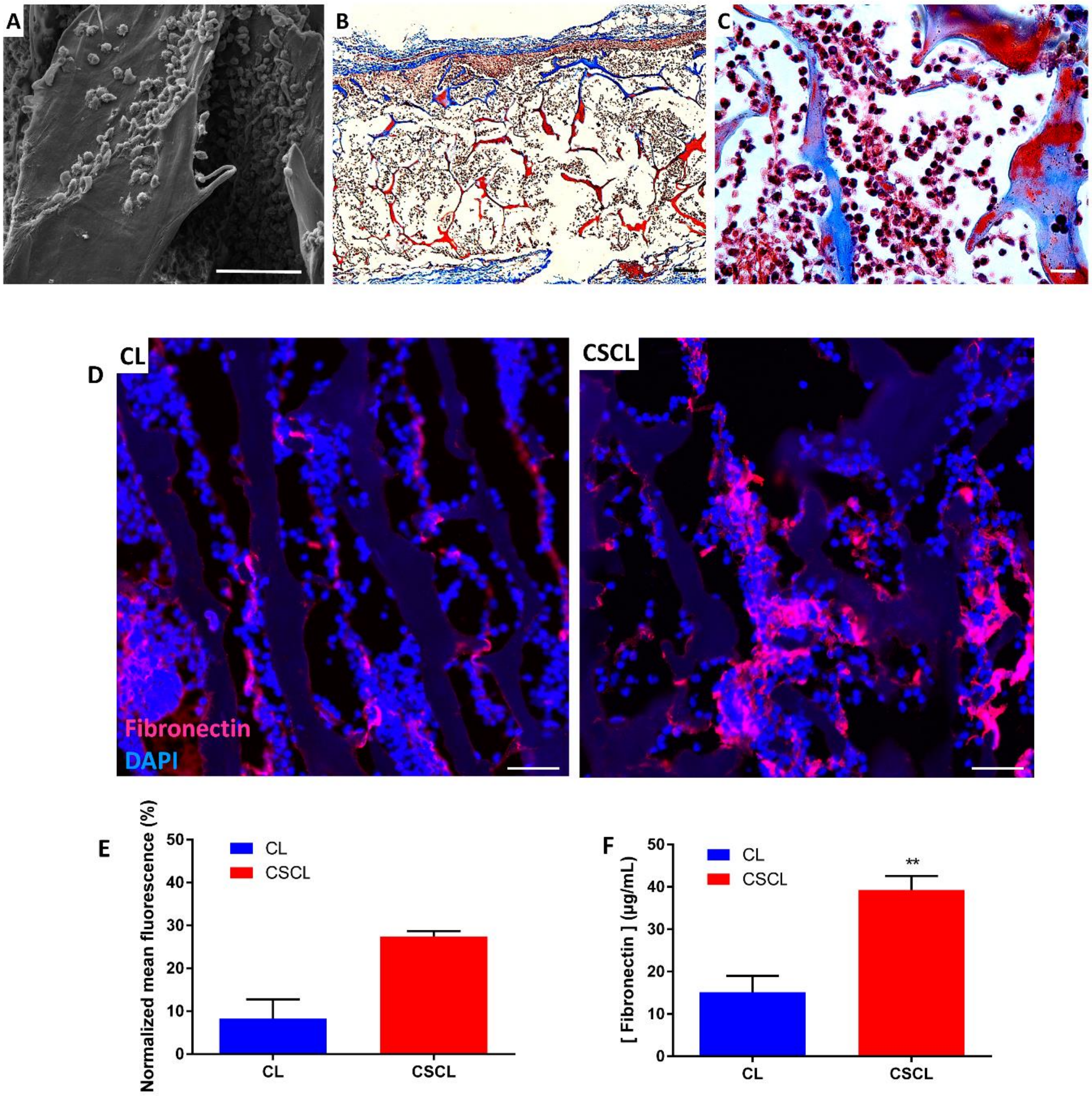


**Figure S1. Infiltrating cells after 1 day from implantation in CL scaffold. A**) SEM evidence the amount of cells that infiltrate the collagen scaffold along collagen thickness. **B**

and **C)** Representative Masson's stained sections at different magnifications. (Scale bars: 200μm, 10μm and 40μm, respectively) **D)** Representative immunofluorescence consecutive sections showing fibronectin presence at 1-day post implant respectively on CL and CSCL. Cells are counterstained with DAPI (scale bars 50 μm). **E)** Quantification of fibronectin immunofluorescence stained section. **F)** Quantification of fibronectin on different scaffold lysate by ELISA assay.

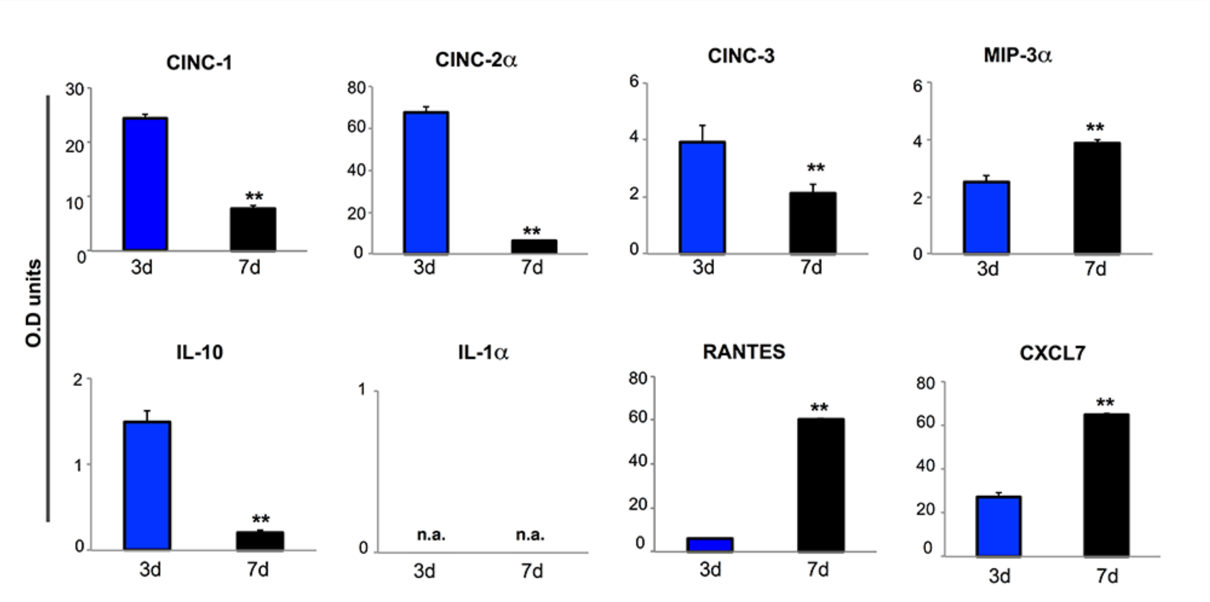


**Figure S2.** Rat cytokines/chemokines profiling of proteins adsorbed onto CL scaffolds at 3 and 7 days post implant. Values are presented as the mean ± SD (n=3, ** $p \leq 0.001$).

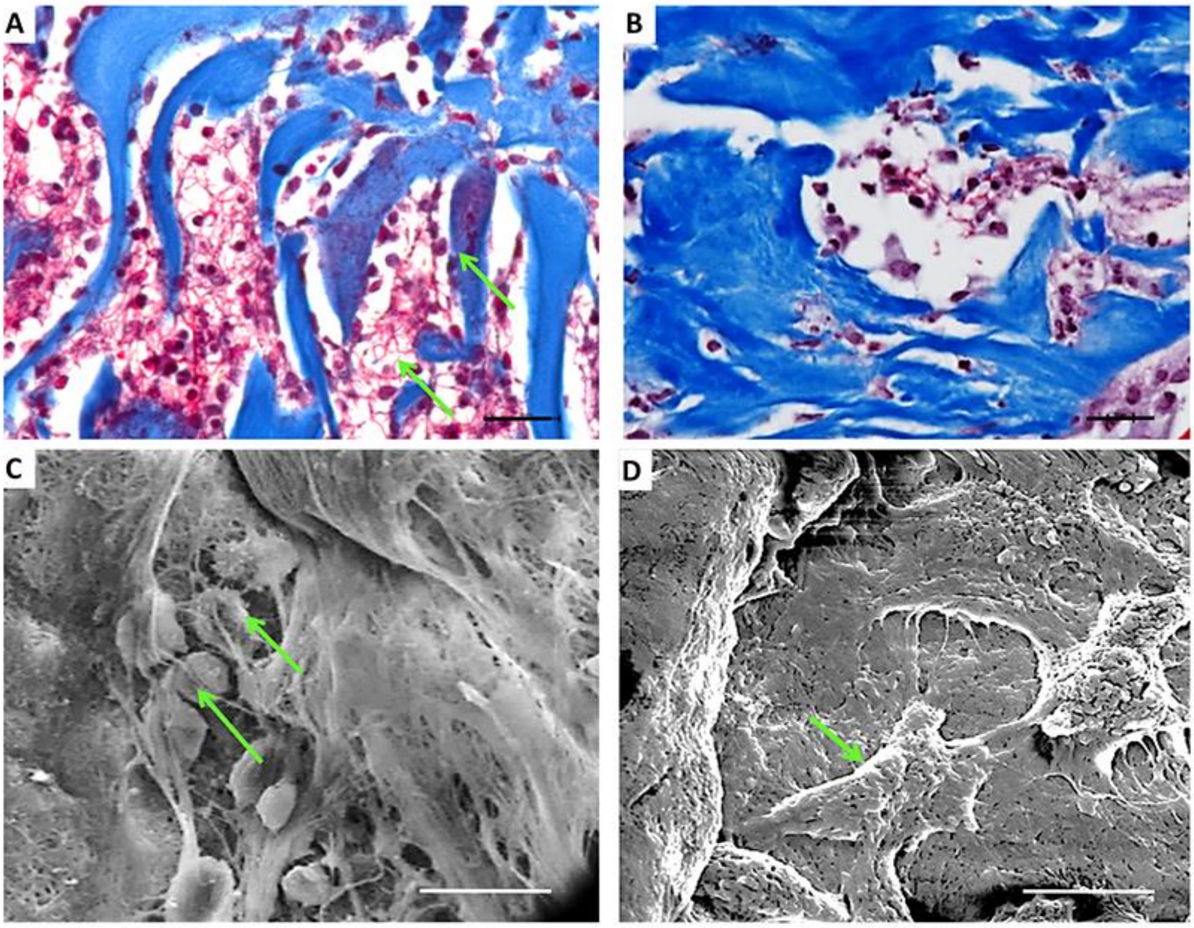


**Figure S3.** Representative Masson's staining and SEM images of CL (**A** and **C**) and CSCL (**B** and **D**) at 7 days. The massive presence of cells and fibronectin is visible in CL, while in CSCL the extracellular matrix is remodeled. Scale bars: histology 40μm, SEM 15μm.

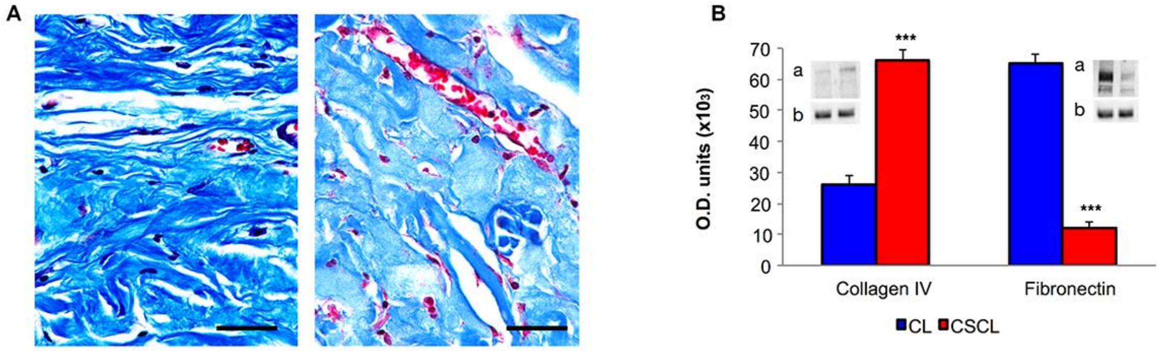


**Figure S4. A**) Representative histological section used for the analysis evidences the presence of big vessels inside and surrounding CSCL scaffold (on the right). Magnification (40x). **B**) Evaluation of fibronectin and collagen IV level of expression (a) in CL and CSCL scaffolds against the control (b, GAPDH). Values are presented as the mean ± SD (n=3, ** $p \leq 0.001$).
